# Width-controlled M-type hexagonal strontium ferrite (SrFe_12_O_19_) nanoribbons with high saturation magnetization and superior coercivity synthesized by electrospinning

**DOI:** 10.1038/srep15089

**Published:** 2015-10-14

**Authors:** Panpan Jing, Jinlu Du, Jianbo Wang, Jinwu wei, Lining Pan, Jianan Li, Qingfang Liu

**Affiliations:** 1 Key Laboratory for Magnetism and Magnetic Materials of Ministry of Education, Lanzhou University, Lanzhou, 730000, People’s Republic of China; 2 Key Laboratory of Special Function Materials and Structure Design of the Ministry of Education, Lanzhou University, Lanzhou 730000, People’s Republic of China

## Abstract

Width-controlled M-type hexagonal SrFe_12_O_19_ nanoribbons were synthesized for the first time via polyvinylpyrrolidone (PVP) sol assisted electrospinning followed by heat treatment in air, and their chemical composition, microstructure and magnetic performance were investigated. Results demonstrated that as-obtained SrFe_12_O_19_ nanoribbons were well-crystallized with high purity. Each nanoribbon was self-assembled by abundant single-domain SrFe_12_O_19_ nanoparticles and was consecutive on structure and uniform on width. PVP in the spinning solution played a significant influence on the microstructure features of SrFe_12_O_19_ nanoribbons. With PVP concentration increasing, the ribbon-width was increased but the particle-size was reduced, which distributed on a same ribbon were more intensive, and then the ribbon-surface became flat. The room temperature magnetic performance investigation revealed that considerable large saturation magnetization (*M*_s_) and coercivity (*H*_c_) were obtained for all SrFe_12_O_19_ nanoribbons, and they increased with the ribbon-width broadening. The highest *M*_s_ of 67.9 emu·g^−1^ and *H*_c_ of 7.31 kOe were concurrently acquired for SrFe_12_O_19_ nanoribbons with the maximum ribbon-width. Finally, the Stoner-Wohlfarth curling model was suggested to dominate the magnetization reverse of SrFe_12_O_19_ nanoribbons. It is deeply expected that this work is capable of opening up a new insights into the architectural design of 1D magnetic materials and their further utilization.

Among various advanced magnetic nanomaterials, M-type hexagonal ferrites with a general formula of MFe_12_O_19_ (M = Ba, Sr and Pb) have emerged as multifunctional materials for vast scientific and technological interests due to their unsurpassed properties such as low price, large magneto-crystalline anisotropy, high Curie temperature, considerable saturation magnetization and superior coercivity as well as amazing chemical stability and corrosion resistance^1^,^2^,^3^. Strontium ferrite (SrFe_12_O_19_), a classical hard magnetic material discovered in 1950s, has been extensively studied for applications in permanent magnets, microwave devices, modern high-density magnetic memory media used in disk drivers and video recorders and so on^2^,^4^,^5^. Nano-SrFe_12_O_19_ also gets the above-mentioned features belonged to M-type ferrites. Nevertheless, a further improvement of its permanent magnetic performance is still of great significance^2^,^6^. Plenty of work has indicated theoretically and experimentally that magnetic properties of nanomaterials are highly relevant with their microstructures, dimensions, components and preparation methods^2^,^5^,^7^,^8^,^9^,^10^.

Recently, considerable progress in synthesizing of one-dimensional (1D) magnetic nanostructures, such as nanofibers, nanotubes and nanoribbons, has been inspiring researchers to either investigate their magnetic-domain configurations and magnetization reversals or develop their electromagnetic nanoscale device applications^11^,^12^,^13^,^14^. As is known to all, 1D magnetic nanostructures possess remarkable geometrical limitations being comparable to the critical magnetic lengths such as exchange length and domain wall width and can effectively overcome the serious aggregation of nanoparticles^15^,^16^,^17^. It means that 1D permanent magnetic nanostructures may get more remarkable magneto-crystalline anisotropy and shape anisotropy^18^. Hence they are capable of offering more creativity for magnetic media, especially the perpendicular magnetic recording media. Jaya Sarkar has pointed out that nanowire arrays potentially enable generating bit densities in excess of 100 Gbit/in^2^ in 2007^19^. Furthermore, the theoretical recording density of 500 Gbit/in^2^ ~ 1 Tbit/in^2^ may be achieved in the quantum magnetic disks with perpendicular recording pattern^20^. Thus far, a few techniques containing hydrothermal route^21^, electrospinning^2^,^22^ and template approach^23^ have been used to synthesize 1D SrFe_12_O_19_ nanostructures. What needs to be stressed is that electrospinning technique is more simple and flexible for producing continuous 1D nanostructures of various materials by using a high voltage dc source^24^,^25^. The diameter or width of electrospun products can be ranged from tens of nanometers to several micrometers. Hence it has got increased attention for many years in a wide range of biomedical and industrial applications, such as drug delivery^26^, wound dressing^27^, air filtration^28^, water purification^29^, sensors^30^ and among others^31^,^32^. Out of the numerous 1D nanostructures synthesized via electrospinning, interestingly, nanoribbons are slightly different from others (nanotubes and nanofibers) because the former has a rectangular cross section but the later have a round cross section. Moreover, nanoribbons also could be considered as a development by cutting a finite-width slice from the 2D nanosheets. Therefore, it is urgently expected that SrFe_12_O_19_ nanoribbons could perform some more interesting magnetic properties.

For the first time in this content, width-controlled SrFe_12_O_19_ nanoribbons were synthesized via a polymer-sol assisted single-spinneret electrospinning route followed by heat treatment in air. Their chemical component, crystalline structure, morphologies and room temperature magnetic properties were investigated in detail. All of the as-synthesized SrFe_12_O_19_ nanoribbons have considerable saturation magnetization and superior coercivity. Moreover, it demonstrated that the ribbon-width and particle-size can be availably modulated by the PVP concentration in the spinning solution.

## Experimental Section

### Materials

Polyvinylpyrrolidone (PVP, Mw = 1,300,000, Alfa Aesar, USA), strontium nitrate (Sr(NO_3_)_2_, 99.5% purity, Sinopharm Chemcial Reagent Co., Ltd, China), iron nitrate nonahydrate (Fe(NO_3_)_3_·9H_2_O, 98.5% purity, Tianjin Kaixin Chemical Industry Co., Ltd, China) and absolute ethanol (C_2_H_5_OH, 99.7% purity, Rionlon Bohua Medical Chemistry Co., Ltd, Tianjin, China) were analytical grade and used as the raw reagents in this work.

### Preparation of SrFe_12_O_19_ nanoribbons

SrFe_12_O_19_ nanoribbons were synthesized via a polymer-sol assisted single-spinneret electrospinning route followed by heat treatment. A typical preparation is as below. Firstly, 0.018 g of anhydrous strontium nitrate (Sr(NO_3_)_2_) and 0.368 g of iron nitrate nonahydrate (Fe(NO_3_)_3_·9H_2_O) were quickly dissolved in 1.5 g of deionized water. Subsequently, a certain amount of PVP powders (0.4 g, 0.5 g and 0.6 g as needed) and 2.4 g C_2_H_5_OH were added together into the above red nitrate solution under vigorous stirring and equilibrated for overnight to acquire a homogenous viscous solution. Of which, the PVP concentrations of these obtained solutions were about 8.5%, 10.4% and 12.3%, respectively. Secondly, right amount of the obtained spinning solution was transformed to a glass syringe equipped with an ordinary stainless needle (the tip was flat and the inner diameter was about 0.4 mm) for electrospinning. The needle was connected with a positive voltage of 15 kV and the aluminum collector was grounded. The vertical distance between the tip of needle and the collector plane was controlled at about 20 cm. Moreover, the spinning solution was withdrawn at a rate of 0.3 mL/h by a micro-injection pump. The whole electrospinning process was conducted at room temperature (about 25 °C) in air. Thirdly, the collected precursor PVP/SrFe_12_O_19_ nanoribbons were kept in a drying oven for several hours, and then were subjected to annealing at 800 °C for 2 h in a muffle furnace in air, respectively. Then SrFe_12_O_19_ nanoribbons were obtained. The heating and cooling rates were both 1 °C/min. For simplicity, we use the S1, S2 and S3 to label the as-obtained SrFe_12_O_19_ nanoribbons resulted from the spinning solutions with PVP concentrations of 8.5%, 10.4% and 12.3%, respectively.

### Characterization

The morphological and microstructural characterizations of the as-prepared SrFe_12_O_19_ nanoribbons were performed by applying field emission scanning electron microscopy (FESEM, Hitachi S-4800) and transmission electron microscopy (TEM, Tecnai^TM^ G^2^ F30, FEI) equipped with an energy dispersive X-ray spectroscopy (EDX). The element and phase component and crystalline structure were determined using powdered X-ray diffraction (XRD, Analytical X’Pert Pro) with Cu-Kα radiation (*λ* = 0.15406 nm) and high-resolution transmission electron microscopy (HRTEM). Room temperature magnetic properties of the SrFe_12_O_19_ nanoribbons were investigated by using a vibrating sample magnetometer (VSM, Lakeshore 7403, USA).

## Results and Discussion

### The composition and microstructure of the prepared SrFe_12_O_19_ nanoribbons

**Figure 1:**
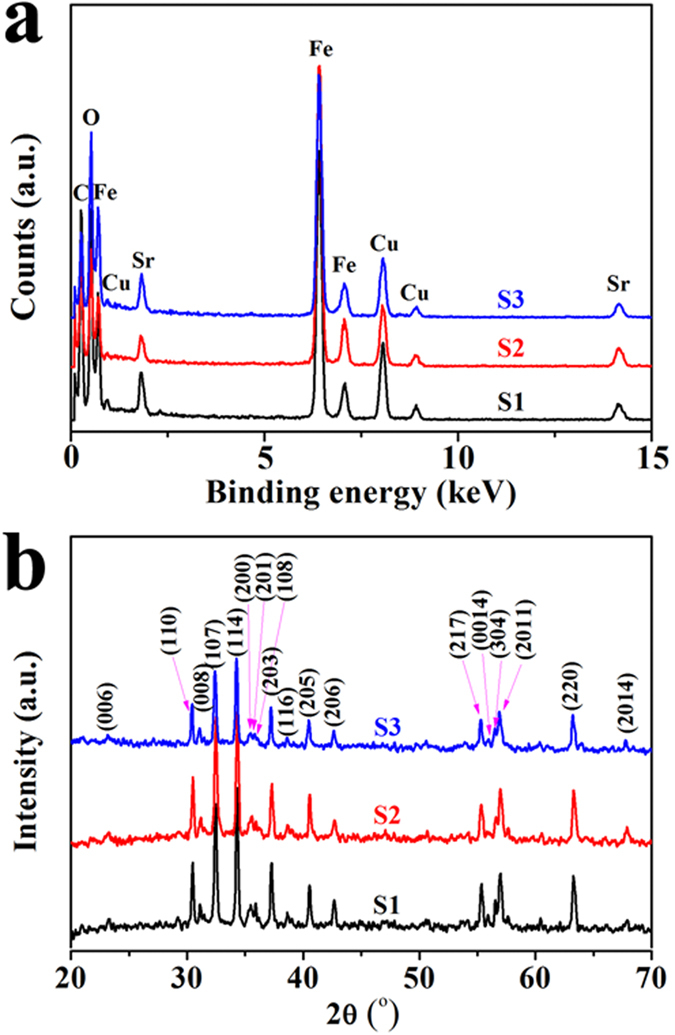
(**a**) EDX and (**b**) XRD patterns of SrFe_12_O_19_ nanoribbons (S1–S3) correspondingly resulted from their spinning solutions with different PVP concentrations (8.5%, 10.4% and 12.3%).

The element and phase component, crystalline structure of as-annealed samples (S1–S3) correspondingly originated from their spinning solutions have been demonstrated by carrying out EDX and XRD analysis. Figure 1a displays their EDX patterns. Targeted elements of Sr, Fe and O are simultaneously detected for all samples. The calculated mole ratios of Sr: Fe are basically equivalent to the stoichiometric ratio of SrFe_12_O_19_. The detected C and Cu elements should be ascribed to the carbon-coated copper grids used for the TEM measurement. Figure 1b shows their XRD patterns. All labeled diffraction peaks in the range of 20^o^ ~ 70^o^ could be well-indexed as (110), (008), (107), (114) crystallographic planes and etc., respectively, and definitely confirm the formation of hexagonal SrFe_12_O_19_ (PDF#33-1340) crystallites with a space group of P63/mmc^2^. It means that all samples are well crystallized into SrFe_12_O_19_ but polycrystalline. No peaks of other additional phases are detected, indicating that these SrFe_12_O_19_ samples are highly purified. Based on these diffraction peaks, the lattice parameters (*a*, *c* and *V*) of S1–S3 are calculated (Table 1) by using the follow equations for the hexagonal crystal system^33^:


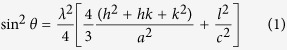



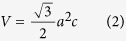


where *θ* is the diffraction angle, *λ* ≈ 1.5406 Å is X-ray wavelength, *h*, *k* and *l* are Miller indexes. The calculated values are basically similar to the cell parameters (*a* = 5. 869 Å, *c* = 23.007 Å and *V* = 686.307 Å^3^) of SrFe_12_O_19_ nanoparticles reported by R. K. Sahu^33^. It is also observed that there is a slight line broadening of some characteristic peaks from S1 to S3, which indicates a reduction in grain size. Using Debye-Scherrer formula^34^:


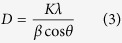


where *K* = 0.9 is a constant, *λ* ≈ 1.5406 Å is X-ray wavelength and *β* is full-width at half-maximum of diffraction peak at 2*θ*, the average crystallite sizes of S1–S3 were also given in Table 1. It suggests that PVP has an influence on crystallization and growth of SrFe_12_O_19_ crystallites in the resultant nanoribbons.

**Figure 2:**
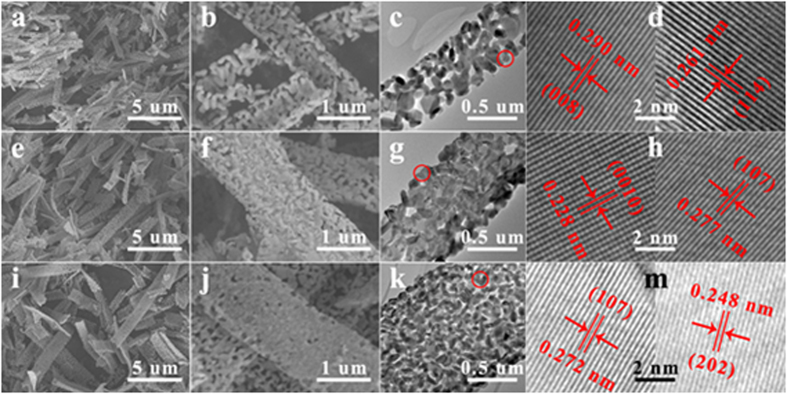
Representative FESEM (the 1^st^ and 2^nd^ columns), TEM (the 3^rd^ column) and HRTEM (the 4^th^ column) images of SrFe_12_O_19_ nanoribbons: (a–d) for S1, (e–h) for S2, (i–m) for S3.

Figure 2 displays the representative SEM, TEM and HRTEM images of the as-prepared SrFe_12_O_19_ nanoribbons (S1–S3). Clearly, as revealed by the low-magnification SEM images (Fig. 2a,e,i), all samples present a novel ribbon-like structure constructed by a large number of interconnecting SrFe_12_O_19_ nanoparticles, which will be further confirmed later. Each nanoribbon is uniform on width and continuous on structure with several micrometers along the long-axis direction. By counting all nanoribbons in the whole SEM image area, the average widths (*W*) are estimated to be about 484 ± 15, 812 ± 10 and 1099 ± 18 nm for S1, S2 and S3 in sequence, indicating that the ribbon-width broadens with PVP concentration increasing in their spinning solutions. Figure 2b,f,j show the large-magnification SEM images of several typical nanoribbons selected from S1–S3. Some pores can be observed obviously between SrFe_12_O_19_ nanoparticles on nanoribbons. It is easy to deduce that these pores are caused by PVP decomposition and SrFe_12_O_19_ nanoparticles crystallization during the heat treatment. For a nanoribbon from S1 to S3, however, these pores shrink gradually and the reduced SrFe_12_O_19_ nanoparticles distribute more and more densely, which make the ribbon-surface smooth and neat. Observation from the typical TEM images for S1–S3 (Fig. 2c,g,k), although these SrFe_12_O_19_ nanoparticles located in the same nanoribbon are not of uniform on size, they have remarkable polyhedron sections. This indicates that all samples have a good crystallinity being in accordance with the XRD results. The average sizes (*d*) of SrFe_12_O_19_ nanoparticles are also estimated to be about 131 ± 3 nm for S1, 94 ± 2 nm for S2 and 76 nm ± 3 for S3, respectively. Figure 2d,h and m show the HRTEM images to further confirm the aforementioned SrFe_12_O_19_ nanoparticles. Of which, lattice fringe distances of about 0.290 nm and about 0.261 nm are severally indexed to the (008) and (114) planes of SrFe_12_O_19_ for S1 (Fig. 2d); lattice fringe distances of about 0.228 nm and 0.277 nm are severally indexed to (0010) and (107) planes of SrFe_12_O_19_ for S2 (Fig. 2h); lattice fringe distances of about 0.272 nm and 0.248 nm are severally indexed to (107) and (202) planes of SrFe_12_O_19_ for S3 (Fig. 2m). Therefore, it is concluded that these novel SrFe_12_O_19_ nanoribbons are self-assembled by abundant SrFe_12_O_19_ nanoparticles, and that PVP concentration in the spinning solutions plays a significant role in controlling their ribbon-width and particle-size.

### The formation mechanism and ribbon-width controlling of the prepared SrFe_12_O_19_ nanoribbons

**Figure 3:**
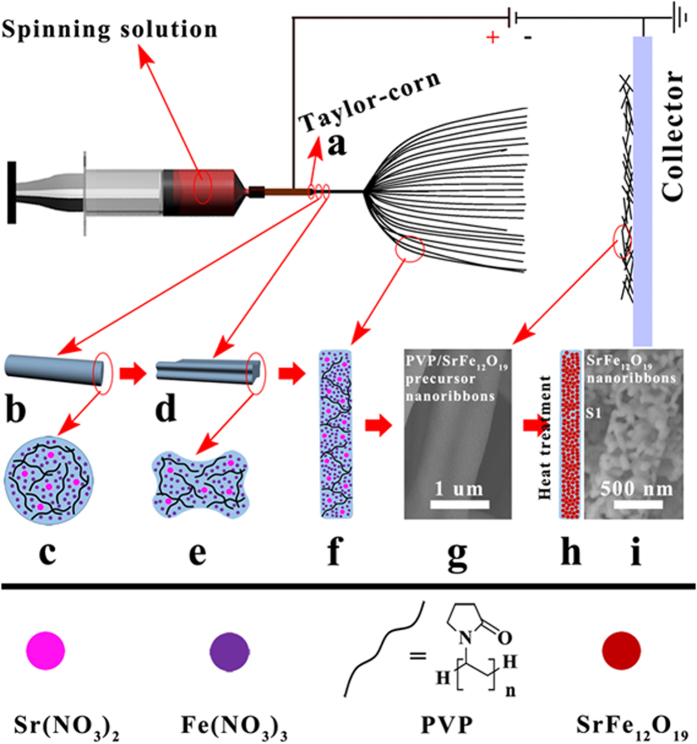
Schematic diagram of the formation mechanism of SrFe_12_O_19_ nanoribbons.

To obtain a visual understanding for the formation mechanism of SrFe_12_O_19_ nanoribbons, a possible schematic diagram is displayed in Fig. 3. The whole preparation can be segmented into electrospinning and heat treatment. Based on the earlier researches, one can be summarized that the novel ribbon-like structure of SrFe_12_O_19_ nanoribbons is produced during electrospinning process^25^,^35^,^36^,^37^,^38^. When the spinning solution arrived up to the spinneret tip, a Taylor-cone shape colloidal droplet (Fig. 3a) formed under the coaction of electrostatic field force and surface tension. With the accumulation of surface charges, the electrostatic field force eventually overcomes the surface tension and a columnar flow-jet (Fig. 3b) with a circular cross section (Fig. 3c) is ejected from the Taylor-cone. Some works have pointed out that the solvents evaporation starts rapidly from the jet surface and causes the jet to become extremely unstable^39^,^40^. So, the PVP concentration of jet surface sol increases sharply and is much larger than that of jet internal sol. If the PVP concentration of the surface sol is increased to a coagulated critical value at a relatively earlier stage, in which the jet travels steadily and extends along a single straight line^41^, the PVP-sol transforms to PVP-gel. Namely, the viscous PVP sol shell begins to freeze and transforms to an elastic skin^38^,^41^. But the internal sol is still viscous flow. Once the elastic behavior of skin overcomes viscous behavior of internal sol, the columnar flow-jet is immediately buckled into a flattened flow-jet (Fig. 3d) with approximately rectangular cross section (Fig. 3e)^38^,^42^. Subsequently, the jet is subjected an unstable stage with a series of bending instabilities and anisotropic shrinkage, and is finally elongated with or without branching/splitting^39^,^40^,^43^ and solidified to PVP/SrFe_12_O_19_ composite precursor nanoribbons (Fig. 3f). Figure 3g shows the SEM image of the collected PVP/SrFe_12_O_19_ composite precursor nanoribbons for S1. When electrospinning is finished, the as-spun PVP/SrFe_12_O_19_ precursor nanoribbons are annealed at the temperature of 800 °C in air, the PVP is degraded completely and the Sr^2+^ and Fe^3+^ ions are compounded to SrFe_12_O_19_. Finally, the SrFe_12_O_19_ nanoribbons (Fig. 3h,i) are constructed by crystalline SrFe_12_O_19_ nanoparticles.

**Figure 4:**
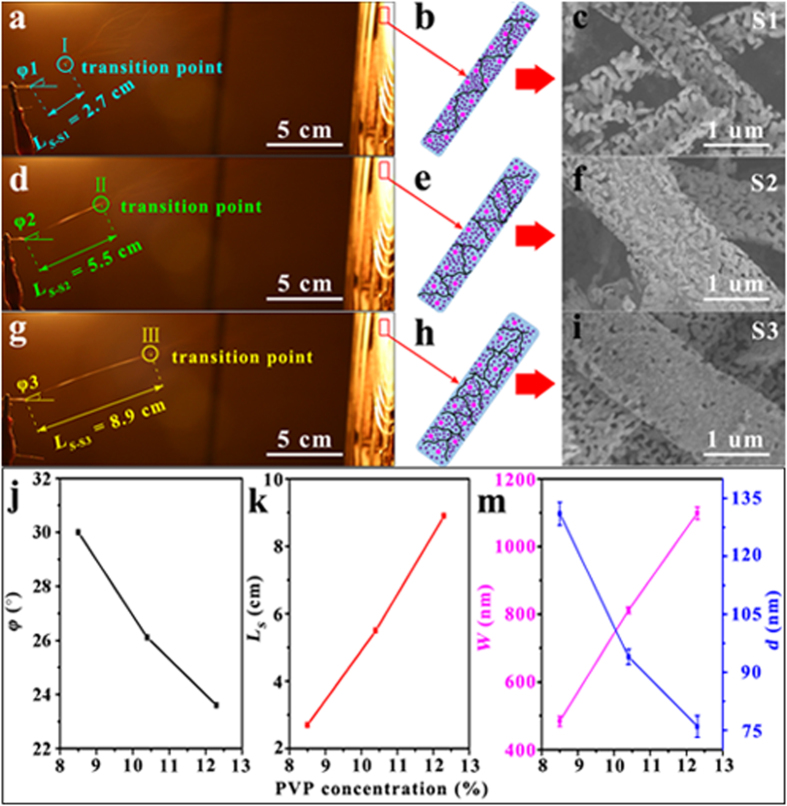
Digital photographs for moments during electrospinning, simulated diagrams for PVP/SrFe_12_O_19_ precursor nanofibers and SEM images of SrFe_12_O_19_ nanoribbons: (a–c) for S1, (d–f) for S2 and (g–i) for S3. The dependences of PVP concentration on the drift angle *φ* between jet initial directions and horizontal direction and length of stable stage *L*_*s*_ during electrospinning for S1–S3 are shown in (**j**) and (**k**), respectively. The variations of average ribbon width *W* and average particle diameter d with PVP concentration are shown in (**m**).

Figure 4a,d,g show the digital photographs grabbed at several stable moments in the spinning process of PVP/SrFe_12_O_19_ precursor nanoribbons for S1–S3, which can be utilized to explore vividly the reason why the SrFe_12_O_19_ nanoribbon width is controlled by PVP concentration in the spinning solutions. Earlier literature has figured out that the electrospun nanofiber diameter mainly depends on the spinneret aperture, polymer (PVP) solution viscosity, feeding rate and voltage supplied^44^. In our work, the unified parameters (a spinneret with an internal diameter of ~0.4 mm, a rate of ~0.3 mL/h and a voltage of ~15 kV) were applied in the electrospinning except the solution viscosity. Hence a higher spinning solution viscosity results in a larger nanoribbon width. When a polymer is dissolved in the mixed solvents of water and ethanol, the solution viscosity is proportional to the polymer concentration^45^. Thus the width of SrFe_12_O_19_ nanoribbons ultimately depends upon the PVP concentration. It should be noted that PVP concentration has a significant influence on the stable stage of electrospinning. Based on the above analysis, besides, the PVP/SrFe_12_O_19_ precursor nanoribbons (Fig. 4b,e,h) are formed at the stable stage of jet traveling and then elongated and narrowed at the unstable stage. Herein, we named a “transition point” at the junction of the stable and unstable stages. For S1–S3, their own “transition points (I, II and III)” are respectively marked by cyan, green and yellow circles. It is observed that the jet directions for S1–S3 at their stable stages were respectively deviated a certain angle (φ) from the horizontal direction and they are measured to be small but noticeable change on the PVP concentration increased from S1 to S3, as revealed in Fig. 4j. During the electrospinning, these drift angles should be ascribed to disturbance resulted from the breakage of equilibrium between the surface intension and electric field force. Of which, the surface intension is always used to overcome the electric field force to maintain the equilibrium of flow-jet. If the PVP concentration is bigger, the surface intension is increased and the equilibrium resisting ability of jet is enhanced and then the drift angle is reduced. In our case, the difference between the largest *φ* for S1 and the smallest φ for S3 is about 6^o^, which can cause a difference between the total jet traveling distances of S1–S3. But the difference is small and could be ignored briefly because the actual motion of jet is very complete. Thus we could assume that the jets for S1–S3 can travel an approximately equivalent distance (*L*) from the tip to collector. For simplicity sake, the *L* can be considered as the sum of lengths of the stable (*L*_s_) and unstable (*L*_us_) stages, i.e., *L* = *L*_s_ + *L*_us_. For S1–S3 with PVP concentration in the jet of about 8.5%, 10.4% and 12.3 wt%, the “transition point” shift gradually towards to collector, and their lengths *L*_*s*_ are measured to be about 2.7, 5.5 and 8.9 cm, respectively. Therefore, *L*_*s*_ is increased with PVP concentration increasing from S1 to S3 (Fig. 4k), i.e., *L*_s*-*S1_ < *L*_s*-*S2_ < *L*_s*-*S3_. In contrast, the length *L*_us_ of unstable stage is reduced correspondingly, i.e., *L*_us*-*S1_ > *L*_us*-*S2_ > *L*_us*-*S3_. Namely, the distance of ribbon elongation and narrowing is reduced from S1 to S3. As a result, the average width of as-spun PVP/SrFe_12_O_19_ precursor nanoribbons (Fig. 4b,e,h) for S1–S3 increases gradually as well as that of the final SrFe_12_O_19_ nanoribbons (Fig. 4c,f,i). For the polymer-sol assisted electrospinning technique, furthermore, polymer PVP molecular chains construct the main frame of the as-spun precursor nanoribbons. And then the metal ions (Sr^2+^ and Fe^3+^ ions) locate in the interspaces among the PVP frame. That is, the PVP frame acts as a stabilizer or a capping for metal ions. When the PVP concentration reaches a certain value, its frame can effectively enable restraining the growth of SrFe_12_O_19_ nanoparticles during annealing^45^. To understand visually, we consider the interspaces between PVP molecular chains as cells for metal ions. A cell is defined as a unit. If the PVP content or concentration is larger, the units for metal ions anchoring are more but smaller and denser (Fig. 4b,e,h). And the nucleation of SrFe_12_O_19_ grains is then more sufficient during annealing. Although these SrFe_12_O_19_ grains are tighter, they can’t excessively swallow each other following as the Ostwald-ripening theory^46^ because of the PVP restraining effect. Therefore, the resultant SrFe_12_O_19_ nanoparticle on a nanoribbon originated from the spinning solution with higher PVP concentration are smaller but distribute intensively, which is just like S3.

### The room temperature magnetic performance of the prepared SrFe_12_O_19_ nanoribbons

**Figure 5:**
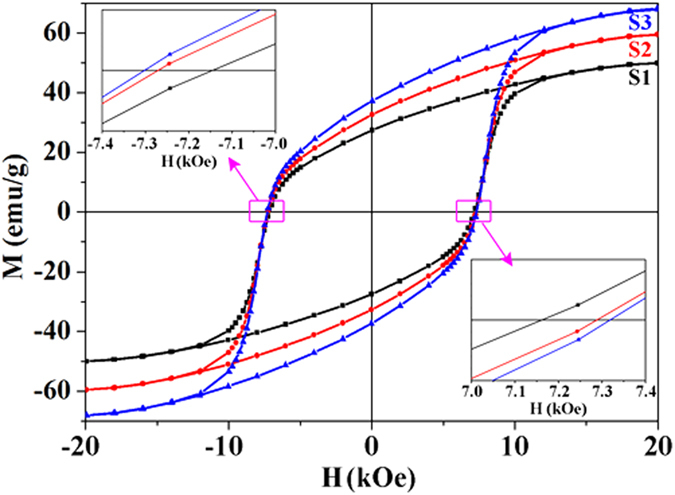
Representative RT magnetic hysteresis (*M-H*) loops of SrFe_12_O_19_ nanoribbons (S1–S3).

Figure 5 shows the *M-H* hysteresis loops recorded at room temperature (RT) to investigate the basic magnetic parameters, such as saturation magnetization (*M*_s_), remanent magnetization (*M*_r_) and coercivity (*H*_c_), of the all SrFe_12_O_19_ nanoribbons (S1–S3). All samples present a typical ferromagnetic behavior. Their calculated *M*_s_, *M*_r_ and *H*_c_ are displayed in Table 2. Of which, the highest *M*_s_ of 67.9 emu·g^−1^ or 346.29 emu·cm^−3^ (*ρ* ≈ 5.1 g·cm^−3^) and *H*_*c*_ of 7.31 kOe for S3 are slightly smaller than the values of 74.3 emu·g^−1^ and 7.4 kOe for theoretical limits predicted by Stoner–Wohlfarth^5^, respectively. Moreover, they are larger than the corresponding reported values of all 1D^2^,^21^,^22^,^23^, several typical 0D^7^,^47^,^48^,^49^,^50^,^51^ and 2D^9^,^10^,^52^,^53^ nanostructures of pure SrFe_12_O_19_ in recent years (Table 2). Their remanence ratios (*M*_r_**/***M*_s_) are basically equivalent and approximately equal to 0.55. Earlier studies have indicated that the single-domain critical size (*D*_c_) for a SrFe_12_O_19_ nanoparticle is about 650 nm^4^, which is much larger than the average sizes (131 ± 3 nm for S1, 94 ± 2 nm for S2 and 76 ± 2.8 nm for S3) of the SrFe_12_O_19_ nanoparticles contained in nanoribbons. Therefore, all nanoribbons consist of abundant single-domain SrFe_12_O_19_ nanoparticles, in which the absence of domain walls and high magneto-crystalline anisotropy result in the magnetization reverse difficulty. And then these SrFe_12_O_19_ nanoribbons have a high *H*_c_. Of course, the unique shape anisotropy also contributes to their *H*_c_ as well as the strong exchange-interaction between SrFe_12_O_19_ nanoparticles and between nanoribbons. Additionally, it is clearly discovered that there is a progressive increase of *M*_s_, *M*_r_ and *H*_c_ from S1 to S3. Just like what has been mentioned in the introduction, although the magnetic properties of assembly nanostructures depend on the particle size and shape of nanoparticles, they are also strongly influenced by the inter-particle interactions^54^. In our case, particularly, the SEM and TEM characterizations have revealed that SrFe_12_O_19_ nanoparticles distribute more and more intensively on nanoribbons from S1 to S3. Thus the amount of magnetic moment per unit volume is increased and then *M*_s_ is increased. Moreover, the exchange-interaction of SrFe_12_O_19_ nanoparticles are increased and then *M*_r_ and *H*_c_ are increased, too^54^,^55^.

The understanding of magnetization reverse is very important to magnetic materials for their technic applications^56^. According to Stoner-Wohlfarth nucleation modes of the micro-magnetism theory, the magnetization reverse mechanism of a nano- or micro-scale system is depended on the coherent radius *R*_coh_. For 1D and 0D magnetic structures, *R*_coh_ = 3.655*L*_ex_ and 5.099*L*_ex_, respectively. For R < *R*_coh_, the magnetization reverse behavior is realized by coherent rotation, whereas for R > *R*_coh_, that is realized by curing^1^,^57^. For a ferromagnetic material such as SrFe_12_O_19_, *L*_ex_ is the exchange length following the equation^17^,^58^:


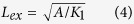


where *A* and *K*_1_ denote the exchange stiffness and the effective anisotropy constant. For SrFe_12_O_19_, *A* = 6.6 × 10^−12 ^J·m^−1^ and *K*_1_ = 3.6 × 10^5^ J·m^−3^ 59. Value of about 4.28 nm is calculated for *L*_ex_ and of about 21.82 nm is calculated for *R*_coh_ for a SrFe_12_O_19_ spherical nanoparticle. In our case, all nanoribbons (S1–S3) are assembled by single-domain SrFe_12_O_19_ nanoparticles, which can be approximately considered as spheres. From the TEM observation, the average radiuses R of SrFe_12_O_19_ spheres are estimated to be about 65.5 nm for S1, 47 nm for S2 and 38 nm for S3, respectively, which are larger than the coherent radius *R*_coh_. Consequently, the magnetization reversal is dominated by the curling model.

## Conclusion

Herein, novel SrFe_12_O_19_ nanoribbons with high crystallinity and purity were successfully synthesized for the first time by using PVP sol-gel assisted electrospinning followed by heat treatment, and they were characterized by EDX, XRD, FESEM, TEM and VSM in detail. Each nanoribbon has been demonstrated to be structural continuous and width uniform and be assembled-well by abundant single-domain SrFe_12_O_19_ nanoparticles along the long-axis direction. Besides, it was revealed that the PVP concentration in the spinning solution has a significant influence on the ribbon-width, surface-flatness and particle-size of SrFe_12_O_19_ nanoribbons, and the potential mechanism was explored systematically. The recorded *M-H* curves at room temperature showed that large values of *M*_s_, *M*_r_ and *H*_c_ were obtained for all SrFe_12_O_19_ nanoribbons and they were increased with broadening of ribbon-width. Particularly, the highest *M*_s_ *and H*_c_ of about 67.9 emu·g^−1^ and about 7.31 kOe were simultaneously obtained for SrFe_12_O_19_ nanoribbons with the maximum ribbon-width. The high *H*_c_ values are mainly attributed to their single-domain particles, high magneto-crystalline anisotropy and the unique shape anisotropy as well as the exchange-interactions between SrFe_12_O_19_ nanoparticles and between nanoribbons. Finally, the curling model can be proposed to guide the magnetization reverse of SrFe_12_O_19_ nanoribbons.

## Additional Information

**How to cite this article**: Jing, P. *et al.* Width-controlled M-type hexagonal strontium ferrite (SrFe_12_O_19_) nanoribbons with high saturation magnetization and superior coercivity synthesized by electrospinning. *Sci. Rep.* **5**, 15089; doi: 10.1038/srep15089 (2015).

## Figures and Tables

**Table 1:** Lattice parameters and grain sizes (*D*) of the as-prepared SrFe_12_O_19_ nanoribbons (S1–S3).

Sample	Lattice parameters			Grain size
	*a*(Å)	*c*(Å)	*V*(Å^3^)	D (nm)
**S1**	5.872 ± 0.002	23.036 ± 0.005	687.80 ± 0.49	46.3 ± 1.1
**S2**	5.872 ± 0.002	23.005 ± 0.006	687.04 ± 0.47	41.7 ± 0.9
**S3**	5.880 ± 0.002	23.018 ± 0.005	689.24 ± 0.49	39.8 ± 1.4

**Table 2:** RT magnetic parameters of the reported pure SrFe_12_O_19_ nanostructures before and in this work.

Dimensional	Years	Nanostructures	RT magnetic parameters		
			*M*_s_	*M*_r_	*H*_c_
0D	2011^47^	Nanoparticles	54.8 emu·g^−1^	29.52 emu·g^−1^	5.26 kOe
	2012^48^	Nanoparticles	58.7 emu·g^−1^	28.7 emu·g^−1^	5.18 kOe
	2012^49^	Nanoparticles	65 emu·g^−1^	32.5 emu·g^−1^	4.3 kOe
	2013^7^	Nanoparticles	64 emu·g^−1^	—	1.8 kOe
	2013^50^	Nanoparticles	60 emu·g^−1^	—	5.2 kOe
	2014^51^	Powders	60 emu·g^−1^	34 emu·g^−1^	6.7 kOe
1D	2004^21^	Nanowires	59.3 emu·g^−1^	—	1.28 kOe
	2010^22^	Nanofibers	64 emu·g^−1^	—	5.21 kOe
	2011^23^	Nanorods	64.5 emu·g^−1^	—	4.94 kOe
	2013^2^	Nanofibers	59 emu·g^−1^	35 emu·g^−1^	6.85 kOe
	**Present**	**Nanoribbons (S1)**	**50** emu·**g**^−**1**^	**27.5** emu**·g**^−**1**^	**7.15** kOe
	**Present**	**Nanoribbons (S2)**	**59.5** emu·**g**^−**1**^	**32.7** emu**·g**^−**1**^	**7.28** kOe
	**Present**	**Nanoribbons (S3)**	**67.9** emu·**g**^−**1**^	**37.3** emu**·g**^−**1**^	**7.31** kOe
2D	2011^52^	Thin films	267 emu·cm^−3^	134 emu·cm^−3^	4.3 kOe
	2012^53^	Thin films	299 emu·cm^−3^	—	2.5 kOe
	2013^10^	Thin films	276 emu·cm^−3^	130 emu·cm^−3^	4.79 kOe
	2013^9^	Thin films	215 emu·cm^−3^	134 emu·cm^−3^	6.63 kOe
